# TbARF1 influences lysosomal function but not endocytosis in procyclic stage *Trypanosoma brucei*

**DOI:** 10.1016/j.molbiopara.2007.06.009

**Published:** 2007-10

**Authors:** Helen P. Price, Meg Stark, Barbara Smith, Deborah F. Smith

**Affiliations:** a Immunology and Infection Unit, Department of Biology, University of York, Heslington, York YO10 5YW, UK; b Technology Facility, Department of Biology, University of York, Heslington, York YO10 5YW, UK

**Keywords:** Arf, ADP ribosylation factor, BSF, bloodstream form, PCF, procyclic form, RNAi, RNA interference, *T. brucei*, *Trypanosoma brucei*, *Trypanosoma brucei*, ARF1, Endocytosis, Lysosome

## Abstract

The ADP ribosylation factors (Arfs) are a highly conserved subfamily of the Ras small GTPases with crucial roles in vesicle budding and membrane trafficking. Unlike in other eukaryotes, the orthologue of Arf1 in the host bloodstream form of *Trypanosoma brucei* is essential for the maintenance of endocytosis. In contrast, as shown in this study, knockdown of TbARF1 by RNA interference has no effect on fluid-phase endocytosis in the insect stage of the parasite. The protein remains essential for the viability of these procyclic cells but the major effect of TbARF1-depletion is enlargement of the lysosome. Our data indicate that protein trafficking and lysosomal function are differentially regulated by multiple factors, including TbARF1, during progression through the *T. brucei* lifecycle.

## 1 Introduction

The secretory system plays a critical role in the efficient sorting and transport of a range of products to the surface of all eukaryotic cells. The small GTPase Arf1 is a vital component of this system, localizing to the Golgi apparatus in yeast and mammalian systems where it has highly conserved functions in vesicular assembly and the activation of phospholipid-modifying enzymes [1]. An exception is the orthologue of Arf1 (TbARF1) that has been recently characterized functionally in the bloodstream form (BSF) of the kinetoplastid parasite *Trypanosoma brucei* [2], a cell type which supports extremely rapid rates of internalisation and recycling of its major outer membrane protein, variant surface glycoprotein (VSG; [3]). Although TbARF1 localizes at or proximal to the Golgi apparatus in these parasites, the primary effect of TbARF1 knockdown is severe inhibition of the endocytic system, leading to cell death. In contrast, overexpression of a constitutively active GTP-bound form of TbARF1 inhibits protein trafficking from the Golgi apparatus to the lysosome [2].

Here we show that the vital role of TbARF1 in endocytosis is not conserved throughout the *T. brucei* lifecycle. In the more sedentary insect procyclic form (PCF) of the parasite, ARF1 is required for cell viability but depletion of the protein by RNA interference (RNAi) has no effect on the uptake of dextran by fluid-phase endocytosis. Instead, the lysosome becomes enlarged, although degradation of the protein p67 within this organelle is not significantly impaired.

## 2 Materials and methods

### 2.1 Disruption of TbARF1 expression by RNAi

The plasmid p2T7ARF1 [2] contains a non-conserved region of the *T. brucei* open reading frame (residues 101–225) between two opposing T7 promoters under the control of tetracycline repressors. Mid-log phase parasites of *T. brucei* procyclic cell line 29–13 [4] were electroporated with 10 μg of *Not*I-digested p2T7ARF1 using methods described previously [5] to produce the stable cell line 29-13/p2T7ARF1. Expression of ARF1-specific dsRNA was induced by incubating parasites in 100 ng/ml tetracycline. Expression of TbARF1 was monitored by quantitative PCR, using SYBR Green Mastermix (Applied Biosystems). Total RNA was extracted from parasites using TRIzol Reagent (Invitrogen), treated with DNase I (Ambion) and reverse-transcribed using Omniscript RT (Qiagen). A 66 bp fragment of TbARF1 was amplified using SYBR Green Mastermix (Applied Biosystems) on a Prism7000 (Applied Biosystems) and compared to levels of a constitutively expressed control, α-tubulin. Oligonucleotides for amplification were: TbARF1 RTF1, 5′-GGCTTCCGCTTTCAAATCC-3′, TbARF1 RTR1, 5′-CATCAAGGCCGACCATAAGAA-3′, TbαTub RTF1, 5′-CGTGAGGCTATCTGCATCCA-3′ and TbαTub RTR1, 5′-CCCAGCAGGCGTTACCAA-3′.

### 2.2 Microscopy

Indirect immunofluorescence assays on parasites were performed as described [6]. Primary antibodies were gifts as follows: rabbit anti-TbGRASP from Graham Warren (Department of Cell Biology, Yale University School of Medicine, New Haven, CT, USA), mouse anti-p67 from Jay Bangs (Department of Medical Microbiology and Immunology, Madison, WI, USA). Primary antibodies were detected using Alexa-Fluor 488 or 633-conjugated secondary antibodies (Invitrogen). Samples were visualized by confocal microscopy using a Zeiss LSM 510 meta with a Plan-Apochromat 63x/1.4 Oil DIC I objective lens and images acquired using LSM 510 version 3.2 software (Zeiss).

For transmission electron microscopy, cells were sequentially treated in 1% (w/v) glutaraldehyde for 1 h, 1% (w/v) tannic acid for 10 min, 0.5% (w/v) osmium tetroxide for 45 min (all in 100 mM phosphate buffer), then in 1% (w/v) aqueous uranyl acetate for 1 h. After dehydration in an acetone series, cells were embedded in Spurrs resin. Sections were cut on a Leica Ultracut, stained with saturated uranyl acetate in 50% ethanol and Reynolds lead citrate, and viewed with a Tecnai 12 BioTwin (FEI) at 120 kV. Images were acquired with a SIS MegaView III digital camera.

### 2.3 Trafficking assays

To monitor the uptake of dextran, cells were resuspended at 5 × 10^7^/ml in 1 mg/ml Alexa-Fluor 488-conjugated dextran (Invitrogen) in SDM-79 medium and incubated for 20 min at 26 °C. For microscopy, parasites were then fixed in 4% paraformaldehyde and co-stained with DAPI, as described previously [7]. For FACS analysis, cells were fixed as above, then resuspended in PBS at a density of 5 × 10^7^/ml. Samples were then divided into two equal aliquots and anti-488 antibody (1:100, Invitrogen) added to one aliquot for 30 min at RT, before washing in PBS. Fluorescence was measured on a Dako Cytomation CyAn flow cytometer using the FL1 detector and results analyzed with Summit v4.1 software.

ER-to-lysosome trafficking and subsequent degradation of the protein p67 was analyzed by metabolic labelling of cells and immunoprecipitation as described previously [8].

## 3 Results

### 3.1 TbARF1 is essential for viability in T. brucei procyclic cells

**Fig. 1:**
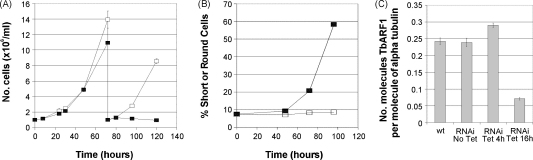
RNA interference in procyclic (PCF) *T. brucei*. (A) growth of cell line 29-13/p2T7ARF1 in the absence (□) or presence (■) of tetracycline over a 6 day time course. (B) % of cells with a round or short (<50% normal body length) phenotype in 29-13/p2T7ARF1 grown in the absence (□) or presence (■) of tetracycline over a 6 day time course. At least 200 cells were scored per sample. (C) quantitative (real time) PCR to measure the number of molecules of TbARF1 transcript per molecule of a constitutively expressed control, α-tubulin, in the 2913 PCF parental cell line (wt) and the transfected cell line 2913/p2T7/ARF1 (RNAi) grown in the absence or presence of tetracycline for up to 16 h.

We studied the functions of TbARF1 in PCF cells by knocking down expression using tetracycline-inducible RNAi. Detection of phenotypic effects arising from TbARF1 knockdown in these cells was considerably delayed in comparison to BSF parasites [2] in which cell death was evident by 24 h post-induction. This probably reflects a lower dependence on rapid protein trafficking in the PCF cell, as observed previously [9]. There was no significant decrease in PCF cell division until 90 h post-induction, when the cells started to become rounded and less motile, followed by cell death from 96 h (Fig. 1A, B). Quantitative PCR showed a significant loss of TbARF1-specific RNA by 16 h post-induction (Fig. 1C). Induced cells had no significant differences in nucleus and kinetoplast configurations between uninduced and induced cells over a 120 h time course (data not shown), indicating no major defects in the regulation of cell division.

### 3.2 Loss of ARF1 has no effect on fluid-phase endocytosis

**Fig. 2:**
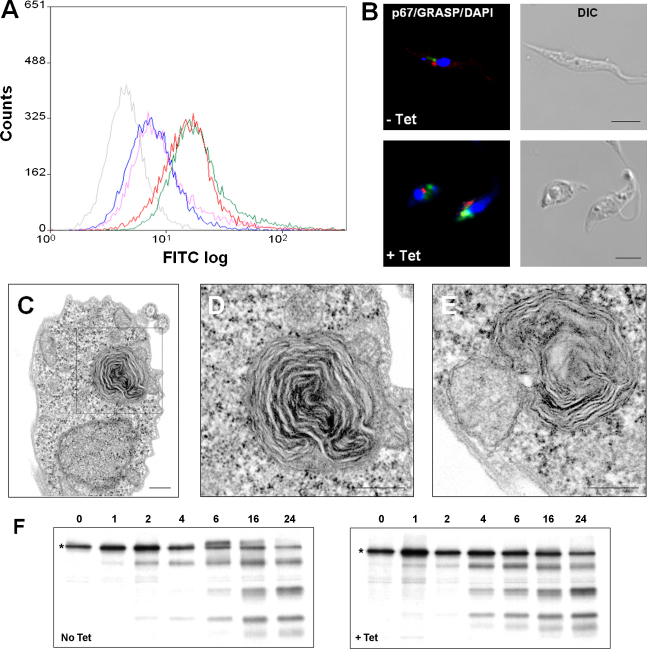
Effects of ARF1 depletion on intracellular trafficking and cell morphology. (A) flow cytometry analysis of the uptake of Alexa-Fluor 488-conjugated dextran by the RNAi cell line 29-13/p2T7ARF1. Uninduced cells + dextran (red); uninduced cells + dextran + anti-488 (blue); cells induced for 96 h + dextran (green); cells induced for 96 h + dextran + anti-488 (pink); cells only (grey). (B) immunofluorescence assays of PCF line 29-13/p2T7ARF1, grown in the absence (−Tet) or presence (+Tet) of tetracycline for 96 h, using antibodies against the lysosomal marker p67 (green) and the Golgi marker GRASP (red) and co-stained with DAPI (blue). Bar, 5 μm. (C, D, E) transmission electron micrographs of cell line 29-13/p2T7ARF1 grown in the presence of tetracycline for 72 h. Image D is an enlarged (x 2.5) view of the boxed area in image C. Bar, 200 nm. (F) immunoprecipitation of p67 from lysates of the cell line 29-13/p2T7ARF1, grown in the absence (No Tet) or presence (+Tet) of tetracycline for 72 h. Metabolic labelling was performed using [^35^S]-labelled methionine and cysteine, with a pulse of 15 min and chase time of up to 24 h. Following immunoprecipitation, captured proteins were separated by SDS-PAGE and products detected by autoradiography. p67 is detected as a 100 kDa protein in the ER (labelled *) and as four major proteolytic fragments following degradation in the lysosome [6].

The effects on fluid-phase endocytosis in these TbARF1-depleted procyclic cells were determined by analyzing the uptake of fluorescently labelled dextran. Analysis by microscopy showed internalization of dextran in both uninduced parasites and cells induced with tetracycline for 96 h (data not shown). We also used a novel FACS-based method to distinguish between external binding and internalization of dextran in these cells (Fig. 2A). Cells were incubated in the presence of Alexa Fluor 488-conjugated dextran, before fixing and incubation in anti-488 antibody which quenches the fluorescence associated with externally bound dextran. No significance differences were observed between uninduced and induced cells, either in total dextran fluorescence or internalized dextran alone (i.e. following antibody treatment) (Fig. 2A).

### 3.3 Loss of ARF1 causes an enlargement of the lysosome

Immunofluorescence assays using the lysosomal marker, p67, showed that TbARF1-depleted procyclic parasites have an enlargement of the lysosome (Fig. 2B). Densitometric analysis of 20 cells per experimental group revealed that p67 staining in cells induced for 96 h covered an area approximately three times as large as in uninduced cells. No differences were seen in the size or position of the Golgi apparatus, as indicated by localization of the GRASP protein (Fig. 2B). The main defect visualized by transmission electron microscopy was the presence of a single electron-dense rosette-like structure near the flagellar pocket in a subset (30%) of cells by 72 h post-induction, before detection of a growth defect in these cells. This structure (Fig. 2C–E) is composed of concentric layers of membrane and resembles that of membranous cytoplasmic bodies (MCBs) found in mammalian cells. These are lysosomal in origin and typically found in sphingolipidoses, a group of lysosomal storage diseases characterized by deficiencies in the lysosomal enzymes required for sphingolipid degradation [10]. Similar structures of mitochondrial origin have also been observed in *T. brucei* following depletion of the glycosomal PEX11-like protein GIM5B [11]. However, we found no obvious differences in mitochondrial structure in procyclic cells following the induction of TbARF1 RNAi, either by electron microscopy or fluorescent staining with Mitotracker (Invitrogen, data not shown). We therefore conclude that the structure detected in Fig. 2 is lysosomal in origin, although proof of this designation requires immuno-electron microscopy with a lysosomal probe.

### 3.4 Effects of ARF1 depletion on lysosomal trafficking and degradation of p67

The trafficking and degradation of p67 was analyzed by pulse-chase metabolic labelling of cells, followed by immunoprecipitation, as described previously [5,8]. In contrast to this process in BSF parasites, ER- synthesized p67 is not further modified in the Golgi in PCF cells, but proteolytic cleavage in the lysosome results in the generation of four glycosylated fragments: gp75, gp42, gp32 and gp28 [8]. In Fig. 2F, proteolytic fragments resulting from lysosomal degradation accumulated in uninduced and induced cells over the same time course, indicating that both trafficking of p67 and its subsequent degradation were unimpaired in cells depleted of TbARF1 protein (Fig. 2F).

## 4 Discussion

The observations presented here and described previously [2] show that TbARF1 is essential for viability throughout the *T. brucei* lifecycle. However, the downstream effects of modulating expression of this protein differ between the two major life cycle stages. In the highly active bloodstream form, TbARF1 is required for both receptor-mediated and fluid-phase endocytosis, and depletion of the protein by RNAi causes a severe defect in these mechanisms, rapidly followed by cell death [2]. In contrast, procyclic cells significantly downregulate the expression of proteins associated with receptor-mediated endocytosis, including clathrin [12], and fluid-phase endocytosis is unaffected by the knockdown of TbARF1 expression. Rather than affecting the endocytic system, the absence of TbARF1 causes enlargement of the lysosome, indicating an accumulation of undegraded and/or mistargeted material.

An interesting finding is that lysosomal targeting of p67 is not disrupted by knockdown of TbARF1 in either of the two life cycle stages but is inhibited by an increase in activated ARF1 protein in BSF parasites [2]. Lysosomal targeting in trypanosomes is not yet fully understood but must differ from higher eukaryotes in several respects. In mammals, most soluble lysosomal enzymes, including acid hydrolases, are sorted using mannose-6-phosphate signals [13] but there is no evidence to suggest that this mechanism is conserved in lower eukaryotes such as the kinetoplastids [8]. An alternative to the mannose-6 receptor, sortilin, has also been implicated in hydrolase sorting [13] but orthologues of this protein are not encoded by the *T. brucei* genome. The remaining class of identified signals, either tyrosine or dileucine-based motifs, are able to bind to GGA and AP adapter proteins, which then trigger the packaging of cargo for clathrin-mediated trafficking from the trans-Golgi network (TGN) to the lysosome. Both GGA and AP proteins are directly recruited to the Golgi apparatus by GTP-bound Arf1 [14]. However, genes encoding GGA proteins and AP-2 are absent from the *T. brucei* genome [2,15,16], implying either a greater dependence on the remaining AP-1 and AP-3 complexes or the operation of additional uncharacterized sorting mechanisms.

Much of our current knowledge of lysosomal targeting in *T. brucei* stems from studies on p67, a protein of unknown function that shares structural similarity but not sequence identity with the mammalian LAMP proteins [17]. This protein is trafficked from the ER via the Golgi to the lysosome, utilizing distinct targeting signals in different stages of the parasite life cycle. In PCF cells, the C-terminal cytoplasmic domain of p67 is necessary and sufficient for lysosomal targeting, whereas this region is not required in BSF cells [8]. While the cytoplasmic domain contains two putative dileucine motifs [8], a recent study has analyzed these regions functionally in PCF and demonstrated that both are required to support maximal targeting [18]. It is likely that p67 is sorted in PCF cells via binding to either AP-1 or AP-3 at the TGN, a process that could be inhibited by knockdown of ARF1. While the data presented here suggest that p67 continues to be targeted efficiently for degradation in the lysosome, this does not preclude a defect in the sorting or trafficking of other essential lysosomal factors.

In BSF parasites, the AP complexes may not be directly involved in p67 sorting, given the redundancy of the dileucine motifs in this life cycle stage [8]. As in PCF cells, knockdown of TbARF1 does not prevent p67 localization to the lysosome [2]. However, in contrast, the inducible expression of a constitutively active form of TbARF1 does not inhibit endocytosis but prevents p67 from reaching the lysosome, resulting in extremely rapid cell death [2]. In this situation, excess ARF1 may be sequestering effector proteins required for alternative pathways which would normally facilitate the correct localization of p67.

Given that forward biosynthetic trafficking to the lysosome is not quantitatively impaired in the absence of ARF1, this protein may alternatively influence the recycling of membranes from the lysosome, a defect that might generate multivesicular-type structures such as those observed at higher resolution in Fig. 2. Further studies are required to pinpoint the relationship between TbARF1 and lysosomal function, analysis which may reveal insights into the secretory pathways of lower eukaryotes.
